# A Plant like Cytochrome P450 Subfamily *CYP710C1* Gene in *Leishmania donovani* Encodes Sterol C-22 Desaturase and its Over-expression Leads to Resistance to Amphotericin B

**DOI:** 10.1371/journal.pntd.0007260

**Published:** 2019-04-03

**Authors:** Ruby Bansal, Shib Sankar Sen, Rohini Muthuswami, Rentala Madhubala

**Affiliations:** 1 School of Life Sciences, Jawaharlal Nehru University, New Delhi, India; 2 Chromatin Remodeling Laboratory, School of Life Sciences, Jawaharlal Nehru University, New Delhi, India; U.S. Food and Drug Administration and Center for Biologics Evaluation and Research, UNITED STATES

## Abstract

**Background:**

*Leishmania donovani* is a protozoan parasite, a primary causative agent of visceral leishmaniasis. Sterol produced via the mevalonate pathway, show differences in composition across biological kingdoms. The specific occurrence of Δ22-unsaturated sterols, containing a double bond at the C-22 position in the side chain occurs in fungi as ergosterol and as stigmasterol in plants. In the present study, we report the identification and functional characterization of a plant-like Cytochrome P450 subfamily CYP710C1 in *L*. *donovani* as the *Leishmania* C-22 desaturase.

**Methodology:**

*In silico* analysis predicted the presence of a plant like *CYP710C1* gene that encodes a sterol C-22 desaturase, a key enzyme in stigmasterol biosynthesis. The enzymatic function of recombinant CYP710C1 as C-22 desaturase was determined. To further study the physiological role of CYP710C1 in *Leishmania*, we developed and characterized an overexpressing strain and a gene deletion mutant. C-22 desaturase activity and stigmasterol levels were estimated in the wild-type, overexpressing promastigotes and heterozygous mutants.

**Conclusion:**

We for the first time report the presence of a *CYP710C1* gene that encodes a plant like sterol C-22 desaturase leading to stigmasterol biosynthesis in *Leishmania*. The recombinant CYP710C1 exhibited C-22 desaturase activity by converting β-sitosterol to stigmasterol. Axenic amastigotes showed higher expression of CYP710C1 mRNA, protein and stigmasterol levels compared to the promastigotes. Sterol profiling of CYP710C1 overexpressing *L*. *donovani* and heterozygous mutant parasites demonstrated that CYP710C1 was responsible for stigmasterol production. Most importantly, we demonstrate that these *CYP710C1* overexpressing promastigotes are resistant to amphotericin B, a drug of choice for use against leishmaniasis. We report that *Leishmania* sterol biosynthesis pathway has a chimeric organisation with characteristics of both plant and fungal pathways.

## Author summary

The cytochromes P450 (P450s) are ubiquitous heme-containing enzymes that affect a vast range of oxidation reactions in nature. Cytochrome P450s (CYPs) play an essential role in the metabolism of endogenous or xenobiotic compounds and steroid. The sterol compositions among biological kingdoms differ in the specific occurrence of Δ22-sterols. The C22-desaturation reaction is catalyzed by independent cytochrome P450 family proteins, CYP61 in fungi, and CYP710 in plants. *Leishmania donovani* is a protozoan parasite that causes visceral leishmaniasis (kala-azar). *In silico* analysis predicted the presence of a plant like *CYP710C1* gene in *L*. *donovani* that encodes a plant like sterol C-22 desaturase, a key enzyme in stigmasterol biosynthesis. Here, we have characterized CYP710C1 protein of *L*. *donovani*. Sterol profile analysis of wild-type, *CYP710C1* overexpressing *L*. *donovani* and heterozygous mutant parasite showed that CYP710C1 is responsible for stigmasterol production. Amphotericin B has been used in India for treatment of visceral leishmaniasis for over a decade. Our results demonstrated that overexpression of *CYP710C1* gene leads to resistance to amphotericin B in *L*. *donovani*. Furthermore, characterization of a plant like *CYP710C1* gene in *Leishmania* indicates the presence of a hybrid pathway that shares a resemblance to both fungal and plant pathways.

## Introduction

Leishmaniasis is caused by an obligate intracellular protozoan parasite of the genus *Leishmania* and is spread by the sandfly vector. The pathological features of leishmaniasis range from self-healing cutaneous lesions to fatal visceral leishmaniasis. Visceral leishmaniasis control relies mainly on chemotherapy due to problems related to vector control and the lack of an adequate vaccine to treat the disease [1]. The development of resistance against currently available antileishmanial drugs has resulted in a growing need for discovering novel drug targets and developing new inhibitors [2].

The cytochromes P450 (P450s) are ubiquitous heme-containing enzymes that affect a vast range of oxidation reactions in nature. Cytochrome P450s (CYPs) are present in all three domains of life and play an essential role in the metabolism of endogenous or xenobiotic compounds and steroids [3–6].

Cytochrome P450 database (http://drnelson.uthsc.edu/CytochromeP450.html) represents the CYP genome (CYPomes) of various species [7]. According to the accepted standard convention, a family and subfamily use a numeral and a letter, respectively. CYP51E refers to the family 51 and subfamily E. Similarly, CYP710A is the family name and CYP710A1-A4 belong to its subfamily. *Arabidopsis* has four genes encoding putative P450 protein belonging to CYP710A subfamily. These are *CYP710A1*, *CYP710A2*, *CYP710A3* and *CYP710A4* [4].

CYP51 is present in fungi and has a role in sterol biosynthesis. Cytochrome P450 CYP61 (sterol C-22 desaturase) is yet another superfamily and is present as CYP710 in plants. CYP710 plant P450s are categorized as putative C-22 desaturase that catalyses the synthesis of stigmasterol and brassicasterol/crinosterol from β-sitosterol and 24-epi-campesterol respectively. Both CYP710A1-A2 and CYP710A4 have been characterized in *Arabidopsis thaliana* and have been shown to have a role in stigmasterol synthesis [4].

Sterols are isoprenoid-derived lipids that are involved in the maintenance of membrane integrity and as biosynthetic precursors of steroid hormones in eukaryotes. The genes encoding enzymes that catalyse the biosynthesis of ergosterol but not cholesterol are present in both *Trypanosoma* spp. and in *Leishmania* spp. [8–10]. This characteristic is more similar to fungi than to other eukaryotes including mammals [11]. The primary component of the *Leishmania* membrane is ergosterol which is functionally related to the maintenance of structural integrity and protection from biotic stress [12]. The enzymes to synthesise cholesterol are not present in *Leishmania*, but they do have detectable levels of cholesterol which they probably take up from their external environment [13]. Plants, on the other hand, have a more complex sterol composition. In plants, β-sitosterol, stigmasterol, campesterol, and cholesterol form the majority of sterols and are present in membranes [14, 15].

**Fig 1:**
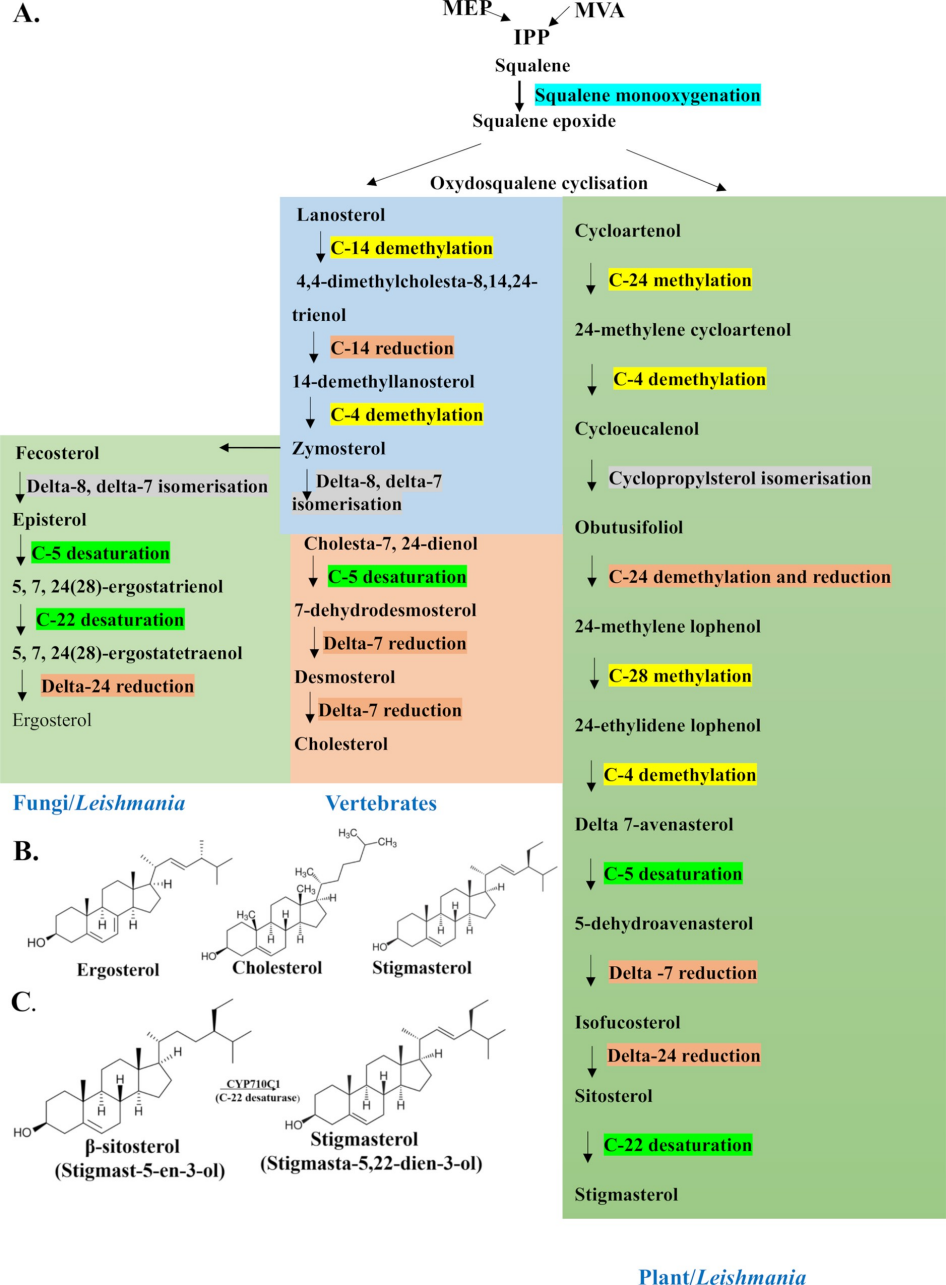
Sterol biosynthesis pathway. A, The canonical pathway of sterol biosynthesis in fungi, *Leishmania*, vertebrates and land plants. B, Structure of ergosterol, cholesterol and stigmasterol molecule with atom numbering. C, Reaction showing the enzymatic conversion of β- sitosterol into stigmasterol in the presence of CYP710C1 (C-22 desaturase).

The sterol biosynthetic pathway has been well-studied in fungi, animals and land plants. Squalene synthesised by mevalonate pathway (MVA-pathway) and methylerythritol-phosphate pathway (MEP-pathway) [16] undergoes step-wise oxygenation and cyclization to form either lanosterol (in vertebrates, fungi and *Leishmania*) or cycloartenol (in land plants) (Fig 1A) [17]. Cycloartenol contains a cyclopropane ring that is subsequently cleaved by plant-specific enzymes in later steps leading to the formation of common sterols with four-carbon rings. In step-wise reactions involving oxidations, reductions and demethylations, lanosterol is converted into cholesterol (in vertebrates) or ergosterol (in fungi and *Leishmania*) while cycloartenol, on the other hand, is converted into phytosterols like campesterol, sitosterol and stigmasterol in land plants (Fig 1A).

The specific occurrence of Δ22-sterols is one of the most spectacular differences in the sterol composition among biological kingdoms [4]. The C-22 unsaturated sterols are primarily found in fungi and plants. For example, ergosterol in fungi and stigmasterol in land plants are Δ22-unsaturated sterols that are structurally different from sterols of animal origin (cholesterol) (Fig 1B). Sterol C-22 desaturase plays an essential role as a terminal enzyme in the sterol biosynthesis in fungi and plants [18]. The C-22 desaturation reaction is catalysed by independent cytochrome P450 family proteins, CYP61 in fungi, and CYP710 in plants. In *Arabidopsis thaliana*, the synthesis of stigmasterol and brassicasterol is catalysed by two separate sterol C-22 desaturases, encoded by the genes *CYP710A1* and *CYP710A2*, respectively [4]. Biosynthesis and physiology of the Δ22-sterols are not clearly understood. *Leishmania* and *Trypanosomes* being non-photosynthetic protozoans synthesise not only lanosterol but also have *CYP710C* (*L*. *major*, XP_001684965) related gene [19, 20]. The presence of *CYP710C* related gene suggests a possible role of this C-22 desaturase in the synthesis of stigmasterol in these parasites (Fig 1C).

The composition of sterols inside fungi and *Leishmania* is a significant determinant of the action of anti-fungal/anti-leishmanial polyene antibiotic amphotericin B (AmB). AmB binds with its mycosamine appendage to ergosterol [21], a significant sterol in fungi, *Leishmania* and *Trypanosomes*. This binding leads to the disruption of membrane integrity coupled with an extensive manipulation of redox balance resulting in the induction of cell death [22]. AmB also causes ergosterol sequestration and induces an oxidative burst, though the exact mechanism of this induction remains mostly unexplored [23]. The AmB can directly interfere with the normal metabolic state of the mitochondria thus influencing the redox state of the cell [24, 25].

We for the first time report the presence of a *CYP710C1* gene that encodes a plant like sterol C-22 desaturase, leading to stigmasterol biosynthesis in *L*. *donovani* [17]. Characterization of a plant like CYP710C1 protein in *Leishmania* was performed. *L*. *donovani* overexpressing *CYP710C1* and heterozygous mutant parasite showed that CYP710C1 is responsible for stigmasterol production. Overexpression of *CYP710C1* gene resulted in resistance to AmB in *L*. *donovani*.

## Materials and methods

### Materials

All restriction enzymes and DNA modifying enzymes were obtained from New England Biolabs (MA, USA). Plasmid pET-30a was obtained from Novagen (Germany). *Escherichia coli* DH10*β* and BL21 (DE3) were used as the host for plasmid cloning and protein expression, respectively. Nickel-nitrilotriacetic acid-agarose was purchased from Qiagen (USA). DNA and protein markers were acquired from New England Biolabs, β- NADPH, β-sitosterol and stigmasterol were acquired from Sigma-Aldrich (USA). Other materials used in this study were of analytical grade and were commercially available.

### Strains and culture conditions

*L*. *donovani* Bob (*Ld*Bob/strain/MHOM/SD/62/1SCL2D) was obtained from Dr Stephen Beverley (Washington University, St. Louis, MO). Wild-type *L*. *donovani Bob* (WT) promastigotes were cultured at 22°C in M199 medium (Sigma-Aldrich, USA) supplemented with 100 mg/ml of penicillin (Sigma-Aldrich, USA), 100 mg/ml of streptomycin (Sigma-Aldrich, USA), and 5% heat-inactivated fetal bovine serum (FBS; Gibco/BRL, Life Technologies, Scotland, UK). WT parasites were routinely cultured in M199 media with no drug supplementation whereas the genetically manipulated heterozygotes (*CYP710C1*/*NEO*), in which one allele of the *LdCYP710C1* gene has been replaced with neomycin phosphotransferase gene were cultured in 300 μg/ml paromomycin whereas *CYP710C1* overexpressing (CYP710C1 OE) parasites were maintained in 5 μg/ml of blasticidin.

The axenic amastigotes were prepared according to the standard protocol [26]. The late-log promastigotes were adapted in an acidic media (RPMI-1640/25 mM 2-(N-morpholino) ethane sulfonic acid (MES)/pH 5.5), at 26°C. These parasites were then grown in RPMI-1640/MES/pH 5.5 at 37°C with 5% CO_2_.

### Expression and purification of the recombinant CYP710C1 protein

The gene for *CYP710C1* (LDBPK_303610) was amplified by PCR using forward primer with a flanking BamHI site (5’ TTTGGATCCATGGCGAAGAAGAAGAAGAAATTCAAGATGGCT -3’) and reverse primer with a flanking HindIII site (5’—TTTAAGCTTCTACACCTTCTCAGCCTTGGGTT -3’) from *L*. *donovani* genomic DNA. The CYP450s are heme containing proteins. Since the standard laboratory strains of *E*. *coli* have a limited capacity to take up heme from the extracellular environment, therefore, several strategies, have been developed for the expression of active CYP450s in *E*. *coli* [27]. One such strategy is N-terminal modification and co-expression of auxillary protein. In the present study, we have used both N-terminal modification and addition of auxillary protein (Cytochrome P450 reducatse) in the enzyme assay. The highest expression levels were observed from the expression constructs that were modified by eliminating a stretch of hydrophobic residues and replacing the first 41 residues with the fragment MAKKKK. The 1527-bp amplification product encompassing the entire *CYP710C1* open reading frame (ORF) was cloned into the pET-30a vector (Novagen) using BamHI and HindIII restriction sites. This construct containing a His_6_-tag at the N terminus was transformed into the *E*. *coli* BL21 (DE3) strain (Novagen). Protein expression was induced with 1 mM isopropyl *b*-D-thiogalactopyranoside at 28°C for 5 h. Bacterial culture was then pelleted down by centrifugation at 5000 *g* for 10 min, and the cell pellet was suspended in lysis buffer (20 mM Tris-Cl, pH 8.0, 10 mM imidazole, 500 mM sodium chloride, 0.4% Triton X-100, 0.5 mM EDTA, 1 mM DTT, 10% glycerol, 2 mM phenylmethylsulfonyl fluoride and protease inhibitor mixture). The overexpressed protein was purified using Ni^2+^ -NTA-agarose resin (Qiagen) by eluting with increasing concentrations of imidazole. The purified protein was found to be 95% pure as judged by SDS-PAGE.

### Semi-quantitative RT-PCR

Total RNA from approximately 2 x 10^6^ parasites was isolated using TRIZOL reagent (Sigma, USA). RNA was precipitated by phenol-chloroform treatment and dissolved in DEPC treated RNase free water and quantified with spectrophotometric analysis. cDNA was prepared from 4 μg of total RNA using First Strand cDNA Synthesis Kit (Thermo Scientific, USA) and random hexamer priming. The subsequent cDNA was analysed by Quantitative real-time PCR experiments (Q-PCR) using SYBR Green PCR Master Mix (Applied Biosystems, CA, USA). Fold change was determined using the comparative (2^-ΔΔCt^) method. In the comparative or ΔΔCt method of qPCR data analysis, the Ct values obtained from two different experimental RNA samples are directly normalized to a reference gene and then compared. The C_T_ value is inversely proportional to the abundance or relative expression level of the gene of interest. In the present study, the reference gene was kinetoplast minicircle DNA specific for *L*. *donovani* with primer sequences as follows: forward primer 5’ CCTATTTTACACCAACCCCCAGT 3’ [JW11] and reverse primer 5’ GGGTAGGGGCGTTCTGCGAAA 3’ [JW12].

### Analysis and extraction of sterols by GC-MS

Late log phase promastigotes (10^9^ parasites) or axenic amastigotes were taken and washed with PBS. The resulting cell pellet was resuspended in 20 ml of dichloromethane: methanol solvent (2:1 v/v), mixed vigorously and incubated for 24 h at 4°C. After centrifugation at 11,000 x g for 1 h at 4°C, the extract was evaporated under vacuum. The extract was saponified with 30% KOH in methanol at 80°C for 2 h. Sterols were then extracted using n-hexane and evaporated. The dried residues were dissolved in dichloromethane. Two volumes of N,O-bis (trimethylsilyl)trifluoroacetamide (BSFTA) was added to an aliquot of the extracted sterol solution, and the tubes were sealed and heated at 80°C for 1 h.

The sterols were subjected to gas chromatography/mass spectrometry (GC/MS) analysis using Shimadzu TD 1020 GC Mass Spectrometer QP2010 Plus that was equipped with DB5 columns (dimethylpolysiloxane/diphenyl ratio, 95/5; dimension 30 m by 0.25 mm). The gas carrier was helium. For analysis, the column was kept at 270°C, the injector and detectors were kept at 300°C. The linear gradient was from 150°C to 180°C at 10°C/min for methyl esters. MS conditions were 280°C; electronic ionization was at 70 eV and an emission current of 2.2 kV. NIST (National Institute for Standards and Technology) library was used for obtaining the Retention time (RT), mass/charge (m/z) values and peak assignment. The Retention time (RT) for a compound is not fixed as several factors can influence it. The Retention time (RT) depends on the matrix effects of analytes. Therefore, the RT can be different between pure standards and cell lysates containing many kinds of sterols. Sterols structures were identified by reference to relative RT and mass spectra (m/z) [4]. In mass spectrometry, several molecules are ionised by the high energy electron beam and form cations thereby leading to different fragmentation pattern. The same compound can, therefore, have different m/z values.

The concentration of the sterols is calculated by comparing the peak area of the analyte in the sample with the peak area of the standard sterol of a known concentration [28].

### Antibody generation and western blot analysis

Purified recombinant CYP710C1 protein (4 mg) was subcutaneously injected in mice using Freund’s complete adjuvant (Sigma, USA), followed by three booster doses of the recombinant protein (2 mg) in Freund’s incomplete adjuvant (Sigma, USA), at 2-week intervals. The mice were sacrificed after the last booster and serum were collected for western blot analysis. Early log phase wild-type promastigotes, axenic amastigotes, *CYP710C1* overexpressing strain and heterozygous mutant parasites were harvested, and the resultant cell pellets were re-suspended in lysis buffer (10 mM Tris-Cl, pH 7.2, 5 mM DTT, 10 mM NaCl, 1.5 mM MgCl_2_, 0.1 mM EDTA, 0.5% Triton X-100, 0.3 mM phenylmethylsulfonyl fluoride (PMSF)). The cell pellets were lysed by freeze-thaw cycles and sonicated on ice followed by centrifugation at 10,000 *g*.

Total soluble cell extracts of promastigote (40 μg) were fractionated on a 10% SDS-PAGE gel and blotted onto a nitrocellulose membrane using an electrophoretic transfer cell (Bio-Rad). After blocking with 5% BSA, the membrane was incubated for 2 h at room temperature with anti- CYP710C1 antibody (1:3000) generated in mice. In case of recombinant protein, incubation was done with anti- CYP710C1 antibody (1:5000). The membrane was then washed with Tris-buffered saline (TBS) containing 0.1% Tween 20 (TBS-T) and incubated with horse-radish peroxidase (HRP)-conjugated anti-mouse antibody (Cell Signaling Technology number 7076S) (1:5000). The blot was developed using 3, 3 -diaminobenzidine (DAB) tablets (Sigma) or ECL kit (Amersham Biosciences) according to the manufacturer’s protocol.

### Enzyme activity assays

Desaturase activity of rCYP710C1 was determined according to the coupled enzyme assay procedure [4]. The reaction mixture (0.5 mL) consisted of 50 mM potassium phosphate, pH 7.25, recombinant CYP710C1 (100 μg protein/mL), 100 mM NADPH, and different substrate concentrations ranging from 75 to 150 μM of β-sitosterol. In order to monitor cytochrome P450 activity, 0.1 unit/mL of a purified recombinant NADPH-P450 reductase (Cytochrome P450 Reductase) was added to the reaction mixture. To check *in vitro* cytochrome P450 enzymatic activity of cell lysates (100 μg) of WT, CYP710C1 OE and *CYP710C1/NEO* mutants were used along with recombinant NADPH-P450 reductase (0.1 unit/mL). The incubation was done up to 90 min at 30°C. The reactions were stopped by adding 50 μL of 1 N HCl. The reaction products were extracted 4x with an equal volume of ethyl acetate. The ethyl acetate extracts were evaporated to dryness, and then dried residues were dissolved in dichloromethane followed by addition of 2 volumes of BSTFA at 90°C for 1 h. The extracts were completely evaporated in a stream of nitrogen gas and were immediately stored at -80°C. These dried lipid residues were dissolved in hexane and subsequently used for GC-MS analysis.

GC-MS, results are based on retention times of standards and NIST mass spectral library match. The availability of a mass spectral library makes this technique attractive as qualitative analysis can be performed despite the lack of commercially available standards.

### Drug susceptibility assays

The susceptibility profile of the *L*. *donovani* wild-type and transgenic promastigotes to the drugs was determined using MTT [3-(4, 5- dimethylthiazol-2-yl) -2, 5- diphenyltetrazolium bromide] (Sigma) assay [29]. Briefly, log-phase promastigotes (5 × 10^4^ cells/well) were seeded in a 96-well flat-bottomed plate (Nunc) and incubated with different drug concentrations at 22°C. After 72 h of incubation, 10 μL of MTT (5 mg/ml) was added to each well, and the plates were incubated at 37°C for 3 h. The reaction was stopped by the addition of 50 μL of 50% isopropanol and 20% SDS followed by gentle shaking at 37°C for 30 min to 1 h. Absorbance was measured at 570 nm in a microplate reader (SpectraMax M2 from Molecular Devices).

### Generation of molecular constructs for replacement of *LdCYP710C1* allele

All sequences were accessed from TriTrypDB (http://tritrypdb.org/tritrypdb/). For inactivation of the *CYP710C1* gene, a targeted gene replacement strategy based on PCR fusion was employed. The flanking regions of the *CYP710C1* gene (5’UTR and 3’UTR) were amplified and fused by PCR to the neomycin phosphotransferase gene (*NEO*). The 5’UTR (770 bp) of the *CYP710C1* gene was obtained from WT *L*. *donovani* genomic DNA by PCR amplification using primers A and B_Neo_ (Table 1). The *NEO* gene was amplified from pX63-NEO with primers C_Neo_ and D_Neo_. The 3 ‘UTR (590 bp) of the *CYP710C1* gene was obtained from *L*. *donovani* WT genomic DNA by PCR amplification using primers E_Neo_ and reverse primer F (Table 1).

**Table 1:** Primers used for generation of the NEO specific linear replacement cassette fragments.

S.No	*L*.*donovani* / primers	Primer Sequence
1	A	5’ GGCGAGTGATCCTTTCGAGGTT 3’
2	B_NEO_	5’ CAATCCATCTTGTTCAATCATAGGAGTGAACCAGGGGATCA 3’
3	C_NEO_	5’ TGATCCCCTGGTTCACTCCTATGATTGAACAAGATGGATTG 3’
4	D_NEO_	5’ATGAGGTGGCAAAAGACGCGTGTTATTCAGAAGAACTCGTCAAGAAG 3’
5	E_NEO_	5’CTTCTTGACGAGTTCTTCTGAATAACACGCGTCTTTTGCCACCTCAT 3’
6	F	5’ AAGAGCGATAGATACGTGGCTCCA 3’

The 5’UTR of *L*. *donovani CYP710C1* gene was then ligated to the antibiotic resistance marker gene (*NEO*) by PCR using primers A and D_Neo_. This fragment (5’UTR marker gene) was then fused with the 3’UTR using primers A and F, yielding the linear replacement cassette, 5’UTR’-Neo-3’UTR. The fragment was gel purified, and about 2 μg of the fragment were transfected by electroporation into the wild-type *L*. *donovani* promastigotes according to the standard protocol [30]. The transfectants were selected in the presence of 300 μg/ml paromomycin (Sigma-Aldrich, USA). The cells resistant to antibiotic selection were checked by PCR-based analysis for the correct integration of the replacement cassette using primers shown in (Table 2). The genotype of the *LdCYP710C1* mutants was confirmed by Southern blotting analysis using a standard protocol [31]. The constructs were sequenced to confirm the correct orientation and sequence fidelity. Our attempts to delete the second copy of the *CYP710C1* gene were not successful. Hence, we proceeded with the characterization of the heterozygous mutant strain.

**Table 2:** Primers used for molecular characterization of genetically manipulated parasites.

S.No	*L*.*donovani* / primers	Sequence
**1**	Primer 1	5’ AGTGGCAATGTGTGTGGGCTGAGCACG 3’
**2**	Primer 2	5’ GGCGTAGGCGAGAGCCAGGAAAATGACCACTT 3’
**3**	Primer 3	5’ AAGATTCTGTACCTACCCACCCTGTACCCCC 3’
**4**	Primer 4	5’ ACGTGACTCGCGCGAAAACAGCGAGTCGTCC 3’
**5**	Primer 5	5’ ATAGCGTTGGCTACCCGTGATATTGC 3’
**6**	Primer 6	5’ AACACGGCGGCATCAGAGCAGCCGATTG 3’

In order to overexpress *CYP710C1* gene in *L*. *donovani*, 1500-bp *CYP710C1* was amplified using the forward primer 5’-TCTAGAATGGACTACAAAGACGATGACGACAAGATGGCAGCGTTTAGTCGTCTCCTCG-3’ with a flanking XbaI site and the reverse primer 5’- AAGCTTCTACACCTTCTCAGCCTTGGGTTC -3’ with a flanking HindIII site. The amplified DNA fragment *CYP710C1* was cloned into the XbaI-HindIII site of pSP-α-blast-α- (*Leishmania*-specific vector) containing a blasticidin acetyltransferase gene as the selection marker. The recombinant vector pSP72α-zeo-α-*CYP710C1* was transfected by electroporation into wild-type *L*. *donovani* promastigotes according to the standard protocol [30], and selection of transfectants was made in the presence of 5 μg/ml blasticidin (Sigma-Aldrich, USA).

### Ethics statement

6 week old female Swiss Albino mice were used for the generation of anti- CYP710C1 protein antibody. Animal experiments were performed according to the guidelines approved by the Committee for Control and Supervision of Experiments on Animals (CPCSEA), Ministry of Environment and Forest, Government of India. The protocol was approved by the Institutional Animal Ethics Committee (IAEC) of Jawaharlal Nehru University (JNU) (IAEC Code Number: 15/2017).

### Statistical analysis

Graph Pad Prism Version 5.0 was used for the statistical analysis. Data shown are representative of at least three independent experiments unless otherwise stated as n values given in the legend. All the experiments were set in triplicate, and the results are expressed as the mean ± S.D. Student's t-test was employed to assess the statistical significance of the differences between a pair of data sets with a *p-value* of *<* 0.05 considered to be significant.

## Results

### *Leishmania* encodes a plant-like CYP710C1

**Fig 2:**
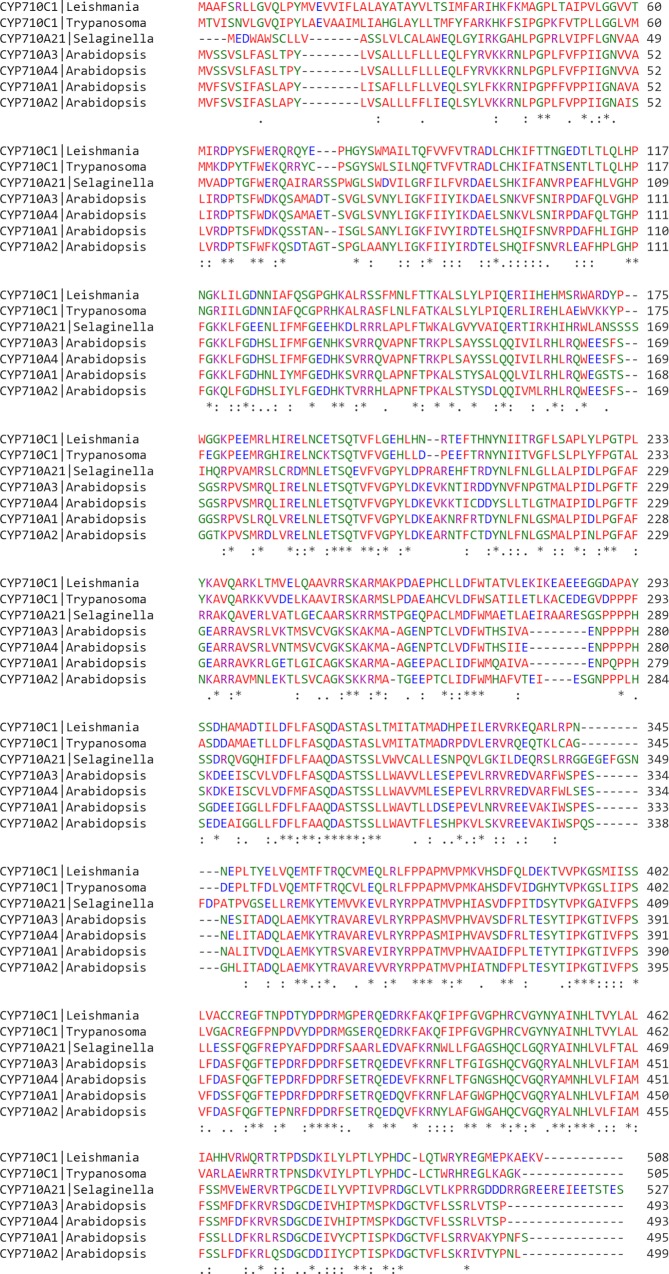
Multiple sequence alignment of CYP710 proteins using Clustal Omega (http://www.ebi.ac.uk/Tools/msa/clustalo/). For analysis, we used the following sequences: *Leishmania donovani* (LdBPK_303610.1), *Trypanosoma cruzi* (TcCLB.510101.50), *Selaginella moellendorffii* (Uniprot ID: D8QPW3) and *Arabidopsis thaliana* CYP710A1 (Uniprot ID: O64697), *Arabidopsis thaliana* CYP710A2 (Uniprot ID: O64698), *Arabidopsis thaliana* CYP710A3 (Uniprot ID:A0A178VR86), *Arabidopsis thaliana* CYP710A4 (A0A178VVZ9). Residues conserved in all five proteins are indicated by an asterisk. Residues having similar, or very similar physiochemical character is indicated by a dot or colon, respectively. The four conserved motifs are highlighted by a rectangular box.

In our earlier report, we have presented an un-rooted phylogenetic tree comparing CYP710, CYP51 of *Leishmania* and CYP51 of *Trypanosoma* with fungi, plant, bacteria and animal counterparts. *Leishmania* CYP710 and plant CYP710 reside in one branch indicating that they are highly similar and are also found to be similar to CYP61 of fungi indicating their common origin [17]. Multiple sequence alignment of different CYP710 with the *Leishmania* CYP710C1 is shown in Fig 2.

Two annotated cytochrome P450 proteins from *Leishmania* with accession number, DQ267494 (CYP5122A1) and UniProt ID: A2TEF2 (X-ray structure available in PDB (http://www.rcsb.org/pdb/) (PDB ID: 3L4D) have been reported in the literature. Our studies identified another cytochrome P450 protein-encoding gene, *CYP710C1*, in the genome of *Leishmania donovani* (UniProt ID: E9BMA4), *Leishmania infantum* (UniProt ID: A4I646), *Leishmania major* (UniProt ID: Q4Q6T3), and *Trypanosoma cruzi CL Brener* (UniProt ID: Q4DX81). This gene is present on chromosome 30 in *Leishmania* while in *Trypanosoma cruzi* it is present on chromosome 32. The *CYP710C1* gene sequence is not present in *Trypanosoma brucei*.

Amino acid sequence alignment of the CYP710C1 protein of *L*. *donovani* with homologous sequences from other strains using Clustal Omega (http://www.ebi.ac.uk/Tools/msa/clustalo/) revealed that CYP710C1 of *L*. *donovani* shares 72% identity with *Trypanosoma cruzi* (CYP710C1), 40% identity with *Selaginella moellendorffii* (Uniprot ID:D8QPW3) (CYP710A21v1) and 38–39% identity with *Arabidopsis thaliana* (CYP710A1-4) (*Arabidopsis thaliana* CYP710A1 (Uniprot ID: O64697), *Arabidopsis thaliana* CYP710A2 (Uniprot ID: O64698), *Arabidopsis thaliana* CYP710A3 (Uniprot ID:A0A178VR86), *Arabidopsis thaliana* CYP710A4 (Uniprot ID: A0A178VVZ9) (Fig 2). The predicated protein sequence of *Arabidopsis thaliana* CYP710A1 is 81.9%, 77.7%, and 76.1% identical to CYP710A2, CYP710A3 and CYP710A4 respectively.

**Fig 3:**
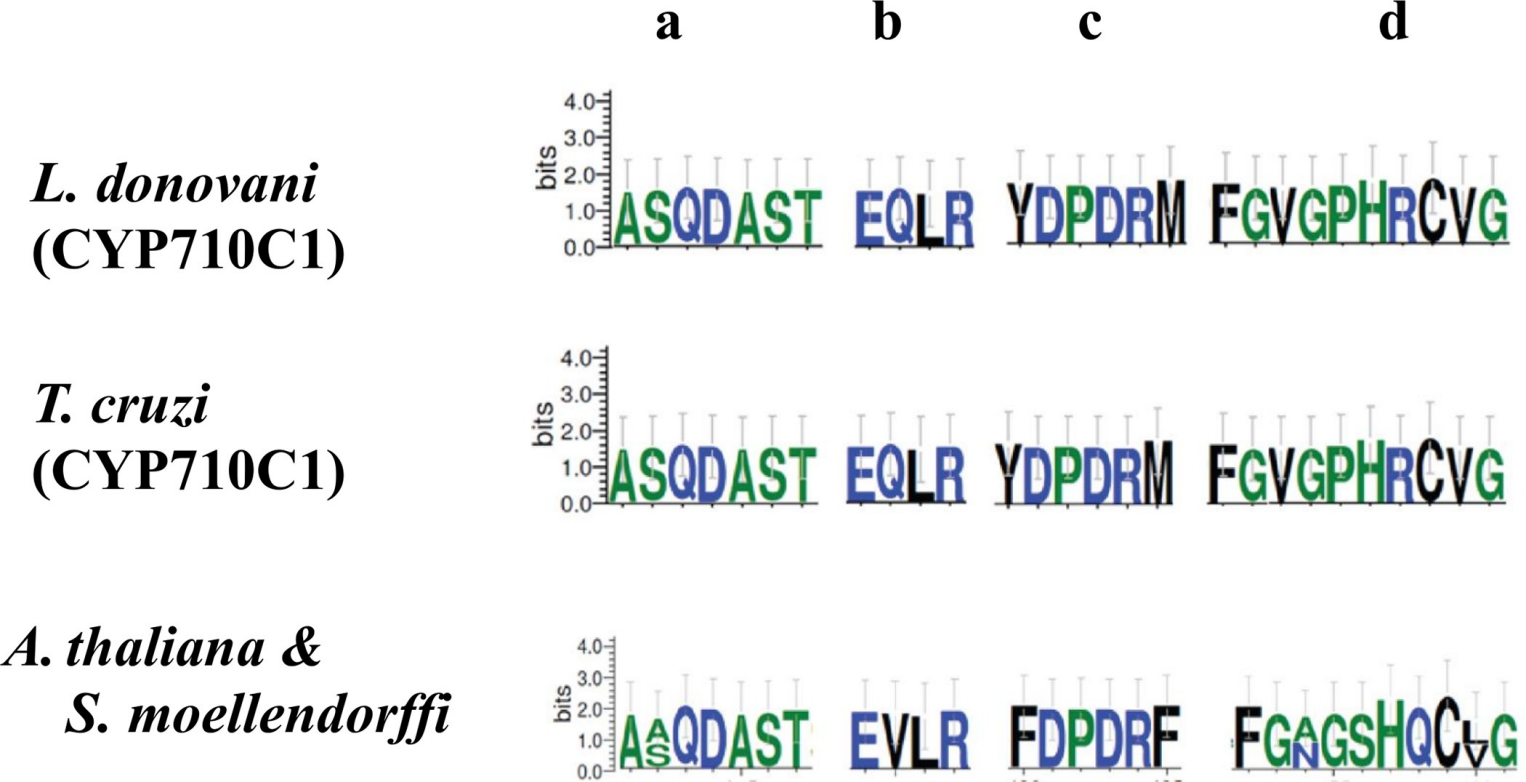
Sequence logos of the conserved CYP motifs of *Leishmania* and their comparison with *Trypanosoma cruzi*, *Arabidopsis thaliana* and *Selaginella moellendorffii*. The most characteristic motif FXXGXRXCXG (d) corresponds to the heme-binding domain. The second conserved motif EXLR (b) and the third consensus PER (c) form E–R–R triad and are essential for stabilization of the core structure. AGXDTT (a) is known to contribute to oxygen binding and activation.

The CYP family of proteins are reported to have four conserved regions. Motif ‘a’ containing the conserved motif AGXDTT contributes to oxygen binding and activation. The second conserved motif EXLR (motif ‘b’) and the third consensus PER (motif ‘c’) form E–R–R triad that is important for locking the heme pocket into position and assuring stabilization of the core structure. Importantly, motif d containing the conserved sequence FXXGXRXCXG corresponds to the heme-binding domain (Fig 3).

Comparison of the sequence logo of all the four conserved motifs of *L*. *donovani* with *T*. *cruzi* and plants (*Arabidopsis thaliana* and *Selaginella moellendorffii*) showed that the proteins show a high degree of conservation. However, there were a few noticeable differences among the motifs (Fig 3). The motif “a” (oxygen binding) showed complete conservation between *L*. *donovani* and *T*. *cruzi* but not between *L*. *donovani* and *S*. *moellendorffii* where in case of plants an alanine is present instead of serine at the second position Motif “b” (EXXR) of *L*. *donovani* was fully conserved and showed similarity with *T*. *cruzi* and plant. The only noticeable difference observed was in the case of plants where the second position had Val in place of Gln. The motif “c” of *L*. *donovani* and *T*. *cruzi* were identical. It also showed similarity with plants except in the first and the sixth position where it has Phe in place of Tyr and Met. Motif “d” of *L*. *donovani* was well conserved and was identical to *T*. *cruzi*. It was also identical to plants except in the third and the ninth position. In the third position, Ala (*S*. *moellendorffii)* and Asn (*A*. *thaliana*) were present in place of Val. In the ninth position, the only difference was in the case of *S*. *moellendorffii* where Leu was present in place of Val. These studies led us to conclude that CYP710C1 from *Leishmania* and plants have highly similar conserved motifs.

### Axenic amastigotes showed higher expression of CYP710C1 mRNA, protein and stigmasterol levels compared to the promastigotes

**Fig 4:**
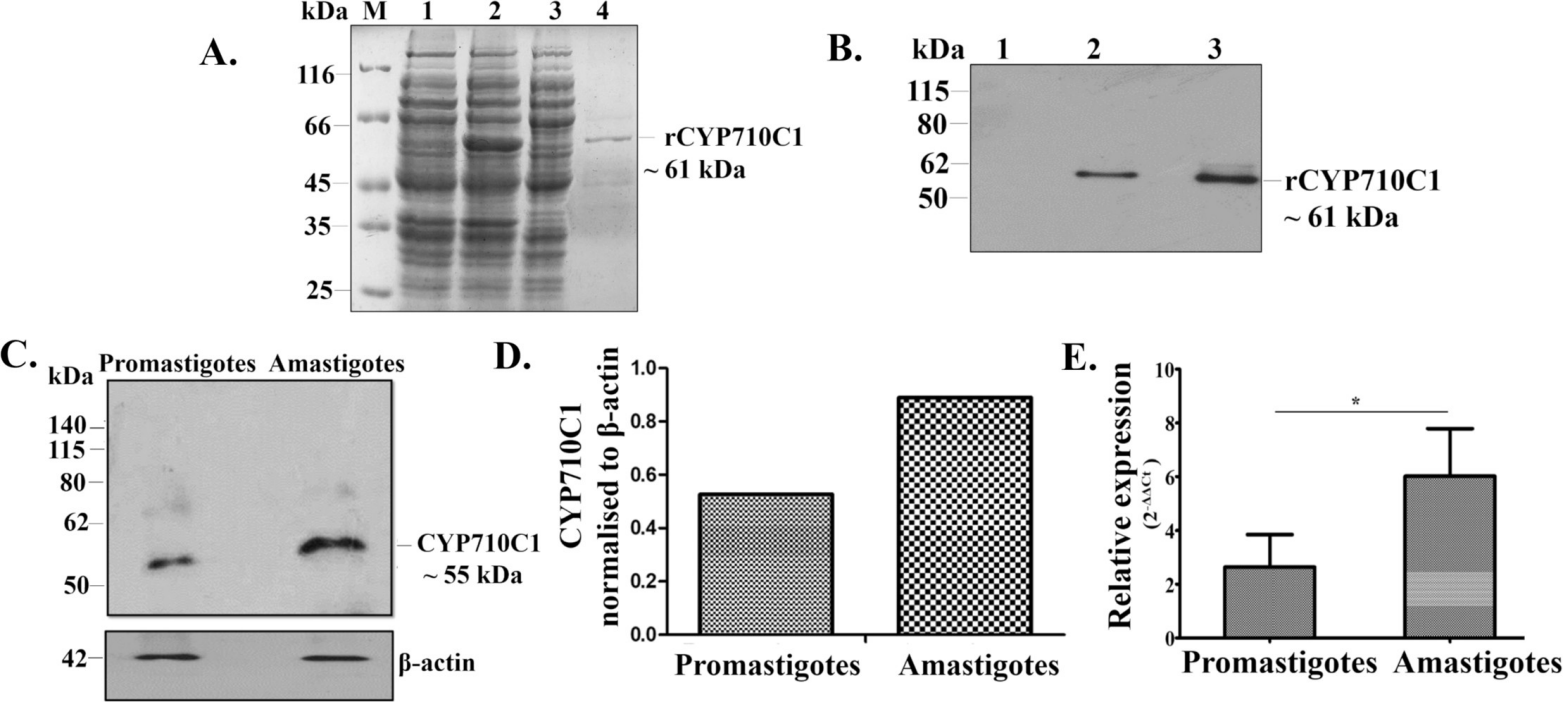
Expression and characterization of recombinant CYP710C1. **A.** The SDS-PAGE analysis of whole cell lysate of uninduced and induced *E*.*coli* BL21 (DE3) cells transformed with pET-30a-*CYP710C1*. M, molecular weight marker; Lane 1 uninduced bacterial cell lysate; Lane 2 induced bacterial cell lysate; Lane 3, Flow through; Lane 4 Purified eluted fraction of recombinant CYP710C1 on Ni^2+^ -nitrilotriacetic acid affinity resin with 100 mM imidazole. **B**, Western blot analysis of recombinant protein using anti-CYP710C1 antibody. Lane 1, negative control, Lane 2, 50 ng rCYP710C1 protein, Lane 3, 100 ng rCYP710C1 protein **C**, Western blot analysis of cell lysates (~ 40 μg) prepared from *L*. *donovani* wild-type promastigotes and amastigotes probed with an anti-CYP710C1 antibody (1:3000). Loading was normalized with β- Actin (42 kDa). **D**, Histogram representing normalized means from densitometric analysis of the immunoblots shown in panel (D) was quantified using ImageJ software. **E**, RT-PCR analysis of CYP710C1 mRNA levels in the cell lysates of amastigotes and promastigotes. The results represent mean ± SD with n = 3. * *p* < 0.05; **, *p* < 0.01, *** *p* < 0.005 and ns indicates not significant (*p* > 0.05).

In order to characterize *L*. *donovani CYP710C1*, the gene was cloned and expressed as His-tagged fusion protein in *E*. *coli* (Fig 4A). The induced His_6_-tagged recombinant CYP710C1 had an estimated molecular size of ~ 61 kDa. The size of rCYP710C1 correlated with the size of the CYP710C1 protein (~ 55 kDa) and His_6_ tag (~ 6 kDa). The recombinant protein was purified to homogeneity by Ni^2+^-NTA metal affinity chromatography (Fig 4A, Lane 4). The expression of the full-length CYP710C1 recombinant protein (~ 61 kDa) was confirmed by immunoblot analysis using anti-CYP710C1 antibody (Fig 4B). The presence of CYP710C1 protein in the promastigotes and the amastigotes was also checked by western blot analysis using anti-CYP710C1 specific antibodies. The anti-CYP710C1 antibody detected a 55 kDa band in the cell extracts of both the promastigotes and the amastigotes (Fig 4C). A higher expression level of the CYP710C1 protein was observed in amastigotes as compared to the promastigotes. A two-fold increase in CYP710C1 mRNA levels was observed in amastigotes in comparison to promastigotes by semi-quantitative RT-PCR (Fig 4E). The C_T_ values of *CYP710C1* and reference gene is reported in Supplementary Information S1 Table. The sterol composition of promastigotes and axenic amastigotes cells was also examined (Table 3). Cell pellets of promastigotes and amastigotes were resuspended in dichloromethane: methanol solvent and incubated for 24 h at 4°C. Sterols were then extracted using n-hexane and evaporated. The dried residues were derivatized and used for GC-MS analysis as reported in the methods section. The major sterols found in promastigotes of *L*. *donovani* were cholesterol (40%) and ergosterol (48%). The axenic amastigotes of wild-type cells contain ~40% cholesterol, ~12.5% ergosterol, ~10% stigmast-5-en-3-ol and ~13% stigmast-5, 22-diene-3-ol of total sterols. The amount of stigmasterol was higher in axenic amastigotes as compared to the promastigotes.

**Table 3:** Comparison between sterols in promastigotes and axenic amastigotes of *L*. *donovani*. Identification is based on matches with the NIST library after fragmentation. Abundance relative to the total sterol content is indicated in each cell line and the fold change of that [32]. Results are the mean % of total sterols ± S.D. of three independent experiments.

S.No	Base Fragment Ion	Molecular Ion (m/z)	Fragment Formula	NIST Library Match	Promastigotes (% of Total)	Amastigotes (% of Total)
1	470	458	C_30_H_54_O	Cholesterol	40.1±1.1	40.1±3.8
2	456	456	C_30_H_52_O	Desmosterol	4.46±0.4	5.25±0.45
3	468	426	C_29_H_46_O_2_	Cholesta-5-7-diene-3-ol	-	5.10±0.5
4	466	396	C_28_H_44_O	Ergosterol	48.01±2.2	12.5±1.2
5	466	396	C_28_H_44_O	Ergosta-5,7, 22-trien-3-ol	3.85±0.3	1.15±0.1
6	470	470	C_31_H_54_O	Ergosta-7,22-diene-ol	-	0.98±0.08
7	470	484	C_32_H_56_O	Stigmasterol	2.06±0.01	2.8±0.4
8	470	414	C_29_H_50_O	β-sitosterol	1.17±0.05	14.97±0.82
9	456	484	C_32_H_56_O	Stigmasta-5,22-diene-3-ol	-	13.77±1.45

### CYP710C1 of *L*. *donovani* is an NADPH dependent desaturase that converts β-sitosterol into stigmasterol

**Fig 5:**
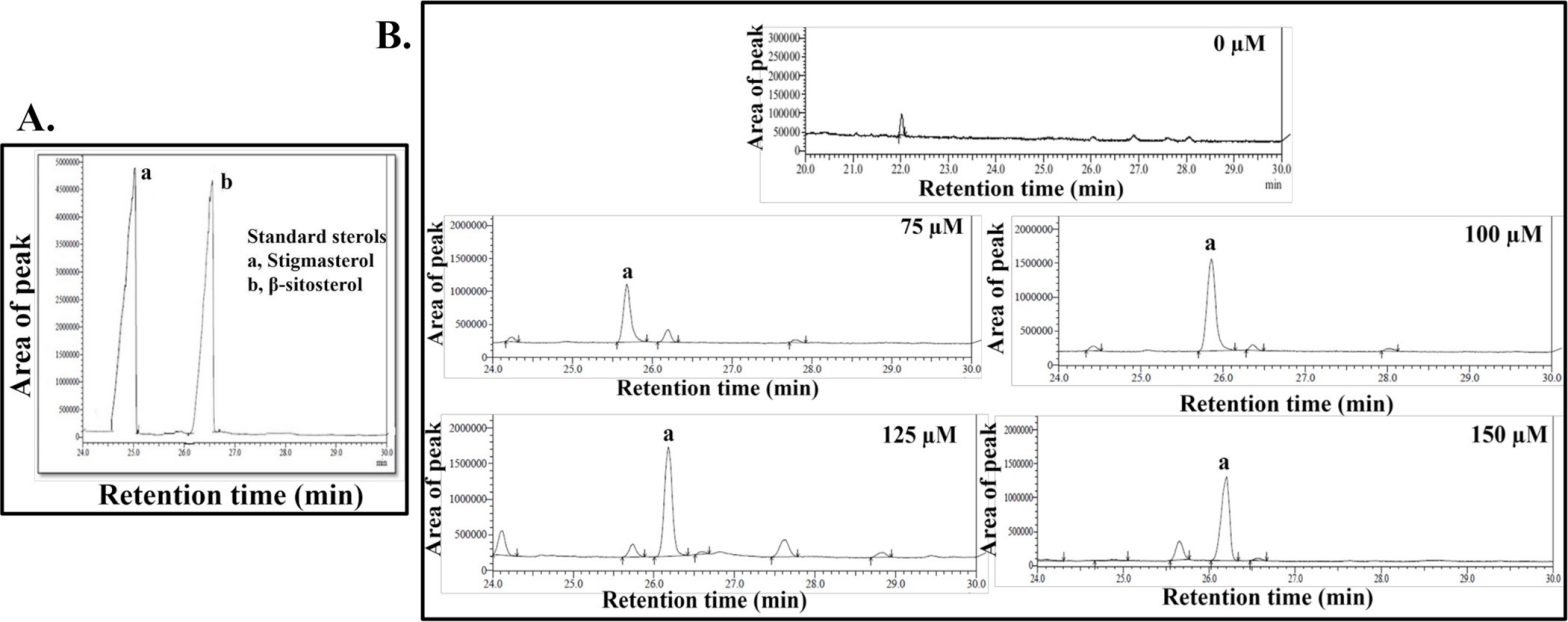
Reaction products from the recombinant CYP710C1 assays were analysed in GC-MS total ion chromatograms. **A** Ion chromatogram of standard sterols at specific retention times (RTs): a, stigmasterol-trimethylsilyl (TMS) (RT = 25.09 min; molecular ion [m/z] = 484) b, β-sitosterol-TMS (RT = 26.2; m/z = 486). **B**, Ion chromatogram showing peak ‘a’ of stigmasterol as Stigmasta-5, 22-dien-3-ol, (Stigmasta-5,22-dien-3-ol is the IUPAC name of stigmasterol, structure is shown in Fig 1) at 0 μM (negative control, where no stigmasterol peak was obtained), 75 μM, 100 μM, 125 μM and 150 μM concentrations of the substrate (β-sitosterol) showing a higher peak at 125 μM concentration. The coupled enzyme assay was done to confirm the desaturase activity of rCYP710C1 (100 μg/ml) with 50 mM potassium phosphate, pH 7.25, 100 mM NADPH, and sterol substrate (β-sitosterol) supplemented with 0.1 unit/mL of a purified recombinant NADPH-P450 reductase (Cytochrome P450 reductase). The pattern of fragment ions with m/z values of 484 and 412 was attributed to stigmasterol whereas 486 and 414 were used to identify β-sitosterol according to the NIST 14 mass spectral library. Three replicates of each reaction show high reproducibility concerning the peak area.

The desaturase activity of rCYP710C1 was assessed using a coupled enzyme assay as explained in the materials and methods. The reaction was carried out with rCYP710C1 in the presence of NADPH and purified recombinant NADPH dependent cytochrome P450 reductase using different concentrations of β-sitosterol, the potential substrate. The gas chromatography analysis of the reaction products from the rCYP710C1 with β-sitosterol as the substrate showed specific peaks at the same retention time (25.09 min) and m/z (484) as that of stigmasterol (Fig 5A) indicating that the CYP710C1 protein catalyzed the C-22 desaturase reaction to produce stigmasterol from β -sitosterol *in vitro*. A concentration-dependent increase in the area of the peak of stigmasterol with varying concentration of β-sitosterol was observed. A maximum increase was observed with 125 μM concentration of β-sitosterol (Fig 5B and Table 4). The results are representative of three different replicates.

**Table 4:** CYP710C1 desaturase activity at different concentrations of substrate (β- sitosterol). Identification is based on matches with the NIST library after fragmentation. Mean values of three replicates are shown.

β-sitosterol concentration (μM)	Retention time (min)	Molecular ion (m/z)	Area of peak	Stigmasterol concentration (μM)	Name of product
75 μM	25.680	484	6,643,685	148 μM	Stigmasta-5,22-dien-3-ol
100 μM	25.699	484	10,407,355	232 μM	Stigmasta-5,22-dien-3-ol
125 μM	26.180	484	15,738,999	351 μM	Stigmasta-5,22-dien-3-ol
150 μM	26.203	484	9,871,721	220 μM	Stigmasta-5,22-dien-3-ol

### CYP710C1 overexpression leads to elevated levels of stigmasterol in *L*. *donovani*

**Fig 6:**
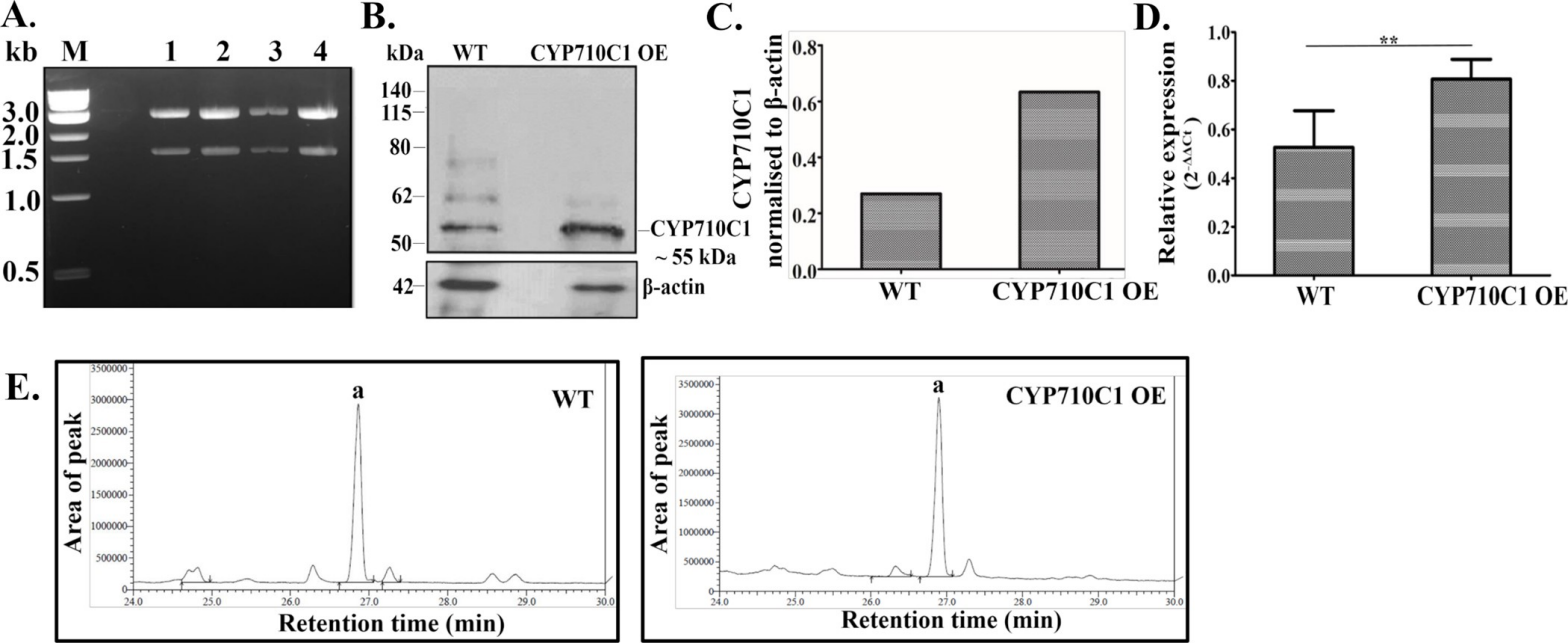
Characterization of CYP710C1 OE. **A,** PCR analysis showing insert fall out confirmation after restriction digestion from the cloned pSP-α-blast-α vector. Lane 1–4, size of *CYP710C1* gene and the vector is 1.5 kb and 4.5 kb respectively **B,** Western blot analysis of protein cell lysates prepared from *L*. *donovani* wild-type Bob promastigotes and CYP710C1 OE probed with the anti-CYP710C1 antibody. Loading was normalized with β- actin (42 kDa). **C**, Histograms representing normalized means from densitometric analysis of the immunoblots shown in panel (B), as quantified using ImageJ software **D**, RT-PCR analysis of CYP710C1 mRNA levels in the cell lysates of CYP710C1 OE and WT promastigotes. The results represent mean ± SD with n = 3. * *p* < 0.05; **, *p* < 0.01, *** *p* < 0.005 and ns indicates not significant (*p* > 0.05). **E**, Ion chromatograms showing peak ‘a’ of stigmasterol after GC-MS analysis of reaction products of CYP710C1 activity by using an equal amount of cell lysate of WT and CYP710C1 OE and with 125 μM of β-sitosterol. Three replicates of each reaction of extraction show high reproducibility about the peak area.

The CYP710C1 encoding sequence was cloned in *Leishmania-*specific pSP-α-blast-α vector (Fig 6A) and transfected into *L*. *donovani*. Both western blot and RT-PCR confirmed that the expression of CYP710C1 was higher in the overexpressing strain (CYP710C1 OE) as compared to the wild-type promastigotes (Fig 6B and 6D). The western blot showed some non-specific bands other than the CYP710C1 (55 kDa) band possibly due to the polyclonal nature of the antibody. Further, cell lysates of CYP710C1 OE promastigotes showed an increased conversion of β-sitosterol into stigmasterol as indicated by enhanced levels of stigmasterol (Fig 6E and Table 5).

**Table 5:** CYP710C1 desaturase activity in WT and CYP710C1 OE by using 125 μM concentration of substrate (β- sitosterol). Representative stigmasterol level as detected by GC-MS analysis of reaction products of CYP710C1 activity in CYP710C1 OE and WT. Identification is based on matches with the NIST library after fragmentation. Mean values of three replicates are shown.

Cell lysate	Retention time (min)	Molecular ion (m/z)	Area of peak	Stigmasterol concentration (μM)	Name of product
WT	26.86	484	2,796,461	62 μM	Stigmasta-5,22-dien-3-ol
CYP710C1 OE	26.89	484	3,024,557	67 μM	Stigmasta-5,22-dien-3-ol

Overexpression of CYP710C1 leads to altered sterol composition in promastigotes and axenic amastigotes of *L*. *donovani*

The sterol compositions of wild-type and CYP710C1 OE *L*. *donovani* promastigotes is reported in Table 6. The significant sterols found in promastigotes of *L*. *donovani* were cholesterol (40%) and ergosterol (48%). In contrast, in CYP710C1 OE promastigotes, the significant sterols were a mixture of cholesterol (~33%), ergosterol (~42%) and stigmasterol (~21%). The possible reason for the peak of cholesterol could be due to the increased uptake from the culture medium as *Leishmania* promastigotes do not synthesize cholesterol but can take it from the surrounding medium [32]. The percentage of stigmasterol in CYP710C1 OE promastigotes was about ten times higher than the wild-type promastigotes (Table 6). We also examined the sterol composition of axenic amastigotes cells derived from the wild-type and CYP710C1 OE *L*. *donovani* promastigotes. The ergosterol contents in axenic amastigotes are low (~12%) compared to wild-type promastigotes. The axenic amastigotes of wild-type cells contain ~42% cholesterol, ~12.5% ergosterol, ~10% stigmast-5-en-3-ol and ~13% stigmast-5, 22-diene-3-ol of total sterols. In contrast, the axenic amastigote of CYP710C1 OE cell lines contained ~67% of stigmasterol and ~17% β-sitosterol-3 acetate. The amount of stigmasterol found in axenic amastigotes was about three times higher than promastigote overexpressing cells and about thirty-four times higher than wild-type promastigotes (Table 7).

**Table 6:** Comparison between sterols in promastigotes of *L*. *donovani* (Bob) wild-type (WT), CYP710C1 OE and heterozygous mutants (*CYP710C1/NEO*). Identification is based on matches with the NIST library after fragmentation. Abundance relative to the total sterol content is indicated in each cell line and the fold change of that. Results are the mean % of total sterols ± S.D. of three independent experiments.

S.No	Base Fragment Ion	Molecular Ion (m/z)	Fragment Formula	NIST Library Match	WT (% of Total)	CYP710C1 OE (% of Total)	*CYP710C1/NEO* (% of Total)	Fold Change (OE/WT)	Fold Change (*NEO/*WT)
1	470	458	C_30_H_54_O	Cholesterol	40.1±1.1	33.24±2.28	23.06±1.4	0.828	0.325
2	456	456	C_30_H_52_O	Desmosterol	4.46±0.4	2.43±0.2	2.24±0.15	0.544	0.502
3	458	428	C_29_H_50_O_2_	Cholest-5-en-3-yl acetate	-	-	5.91±0.48	-	-
4	468	426	C_29_H_46_O_2_	Cholesta-5-7-diene-3-ol	-	0.097±0.001	-	-	-
5	470	386	C_27_H_46_O	Cholest-8-en-3-ol	-	-	10.28±1.0	-	-
6	466	396	C_28_H_44_O	Ergosterol	48.01±2.2	42.19±3.5	52.71±5.58	0.878	1.30
7	466	396	C_28_H_44_O	Ergosta-5,7, 22-trien-3-ol	3.85±0.3	0.02±0.001	2.42±0.24	0.005	0.628
8	470	484	C_32_H_56_O	Stigmasterol	2.06±0.01	21.26±3.2	-	10.32	-
9	482	410	C_29_H_46_O	Stigmasta-4,7,22-trien-3-ol	-	-	1.90±0.12	-	-
10	470	414	C_29_H_50_O	β-sitosterol	1.17±0.05	0.5±0.004	1.51±0.05	0.427	1.29

**Table 7:** Comparison between sterols in axenic amastigotes of *L*. *donovani* (Bob) wild-type (WT), CYP710C1 OE and heterozygous mutants (*CYP710C1/NEO*).

S.No	Base Fragment Ion	Molecular Ion (m/z)	Fragment Formula	NIST Library Match	WT (% of Total)	CYP710C1 OE (% of Total)	*CYP710C1/NEO* (% of Total)	Fold Change (OE/WT)	Fold Change (*NEO*/WT)
1	470	458	C_30_H_54_O	Cholesterol	40.1±3.8	33.24±3.3	45.5±5.5	0.828	1.13
2	456	456	C_30_H_52_O	Desmosterol	5.25±0.45	-	4.8±0.4	-	0.914
3	468	426	C_29_H_46_O_2_	Cholesta-5-7-diene-3-ol	5.10±0.5	5.2±0.5	6.6±0.5	1.019	1.294
4	466	396	C_28_H_44_O	Ergosterol	12.5±1.2	-	22.6±2.5	-	2.1
5	466	396	C_28_H_44_O	Ergosta-5,7, 22-trien-3-ol	1.15±0.1	1.11±0.1	2.82±0.2	0.965	2.45
6	470	470	C_31_H_54_O	Ergosta-7,22-diene-ol	0.98±0.08	-	0.88±0.07	-	0.89
7	470	484	C_32_H_56_O	Stigmasterol	2.8±0.4	67.24±5.84	1.71± 0.15	24.01	0.61
8	470	414	C_29_H_50_O	β-sitosterol	14.87±0.82	17.51±1.8	9.11±0.2	1.17	0.61
9	456	484	C_32_H_56_O	Stigmasta-5,22-diene-3-ol	13.77±1.45	-	5.86±0.48	-	0.42

### Generation of *LdCYP710C1* heterozygous mutants

**Fig 7:**
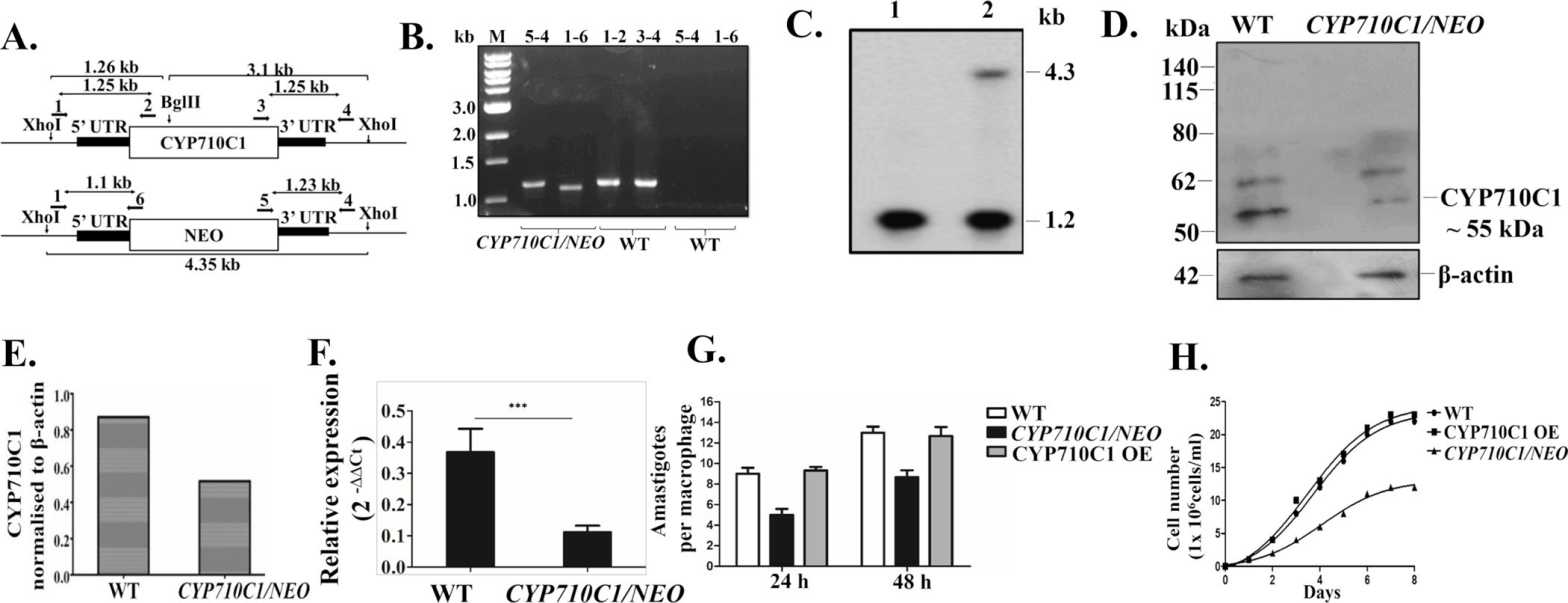
Characterization of *CYP710C1* heterozygous mutants. A, Restriction map of the *LdCYP710C1* genomic locus and location of the primers used for confirmation by PCR-based analysis along with the expected band sizes. B, PCR analysis of heterozygous mutants (*CYP710C1/NEO*) to evaluate the specific integration of the replacement cassette by using NEO and *LdCYP710C1* (WT) gene-specific primers. Genomic DNA from *LdCYP710C1* heterozygous mutants (*CYP710C1/NEO*) and WT parasites was used as a template for PCR analysis. M indicates the molecular size markers in kilobases. Lane numbers in panel B indicate the primers (mentioned in panel A) used for each lane. C, Southern blot analysis of genomic DNA from wild-type (WT) (lane 1), heterozygous *LdCYP710C1* (*CYP710C1/NEO*) (lane 2) mutant parasites. Genomic DNA was digested with XhoI, and BglII separated on a 0.6% agarose gel and probed with the 5’UTR of the *LdCYP710C1* gene. In the lane of WT, digestion of the *LdCYP710C1* gene locus with XhoI yielded a 1.2-kb band whereas in case *CYP710C1/NEO* there was an extra band of 4.3 kb in addition to 1.2 kb. Molecular sizes are shown to the right of the blot. D, Western blot analysis of cell lysates prepared from L. donovani wild-type promastigotes and *CYP710C1* heterozygous mutants (*CYP710C1/NEO*) probed with the anti-CYP710C1 antibody. Loading was normalized with β- actin (42 kDa) E, Histograms representing normalized means from densitometric analysis of the immunoblots shown in panel (D) and in two other experiments, as quantified using ImageJ software F, RT-PCR analysis of CYP710C1 mRNA levels in the cell lysates of *CYP710C1* heterozygous mutants (*CYP710C1/NEO*) and WT promastigotes. G, Infectivity assay in which J774A.1 cells were infected with WT, *CYP710C1/NEO* and CYP710C1 OE parasites. H, Growth curve of WT, CYP710C1 OE and *CYP710C1/NEO* mutants. The results represent mean ± SD with n = 3. *** p < 0.005 the statistical difference from the wild-type control.

The essentiality and function of *LdCYP710C1* in the life cycle of the parasite were determined by replacing one allele of this gene with cassettes harbouring drug-resistance marker gene using targeted gene replacement strategy. This was achieved by generation of inactivation cassettes having neomycin phosphotransferase (*NEO*) as selection markers along with 5’UTR and 3’UTR of the *CYP710C1* gene, as described under Materials and Methods (Primers position marked in Fig 7A). Linear replacement cassettes made by PCR-based fusion were transfected into wild-type *L*. *donovani* promastigotes, leading to the generation of heterozygous parasites. Replacement of a single copy of the *LdCYP710C1* gene was confirmed by PCR-based analysis using primers external to the transfected inactivation cassette of the *CYP710C1* gene. DNA from the transfected *L*. *donovani* promastigotes was isolated and subjected to PCR-based analysis which showed 1.1 (with primer 1 and primer 6) and ~1.2 kb (with primer 5 and primer 4) bands in the case of the *NEO* cassette (Fig 7B). Similarly, a band of ~1.25 kb was observed in the case of WT with WT specific primers (1–2, 3–4) given in Table 2. However, no band was observed when PCR reaction was set up with WT genomic DNA and *NEO* specific primers (5–4, 1–6) confirming that one allele of the WT *LdCYP710C1* gene had been replaced in these heterozygous mutant parasites.

The genotype of the heterozygous (*CYP710C1*/*NEO*) mutant parasites was further confirmed by Southern blot analysis. Genomic DNA from WT and transgenic parasites were digested with the XhoI and BglII restriction enzymes. In the WT cells, digestion of the *LdCYP710C1* gene locus with XhoI yielded a ~1.26-kb band after probing with the 5’UTR of the *LdCYP710C1* gene (Fig 7C). Integration of *NEO* cassette in single transfectants was expected to yield ~4.35 kb 5’UTR hybridizing bands, respectively, in addition to the 1.26 kb wild-type band (Fig 7C). Our attempts to delete the second allele of *CYP710C1* were not successful.

Both western blot and RT-PCR data further confirmed *CYP710C1* heterozygous mutants had lower expression of CYP710C1 as compared to the wild-type promastigotes (Fig 7D and 7F).

The cell lysates (100 μg) of *CYP710C1* heterozygous mutants (*CYP710C1/NEO*) had lower levels of stigmasterol as compared to the cell lysates of wild-type promastigotes (Table 8) leading us to conclude that deletion of one allele of *CYP710C1* led to the decreased conversion of β-sitosterol into stigmasterol. *In vitro* study on the survival of WT, CYP710C1 OE and *CYP710C1/NEO* inside murine macrophages J774A.1was performed. Virulence studies were carried out to detect the effects of genetic deficiency of *CYP710C1* inside murine macrophages. A murine macrophage cell line was infected with WT, heterozygous mutant parasites *CYP710C1/NEO* and CYP710C1 OE at a multiplicity of infection (MOI) of 20:1. WT parasites were capable of infecting and sustaining robust infection in murine macrophages, whereas the parasitemia of the heterozygous mutants was reduced by around 40% relative to that of WT parasites both at 24 h and 48 h post-infection (Fig 7G). The infectivity of CYP710C1 OE was equivalent to WT.

**Table 8:** CYP710C1 desaturase activity in WT and *CYP710C1/NEO* by using 125 μM concentrations of substrate (β- sitosterol). Representative stigmasterol level as detected by GC-MS analysis of reaction products of CYP710C1 activity in WT and *CYP710C1/NEO*. Identification is based on matches with the NIST library after fragmentation. Mean values of three replicates are shown.

Cell lysate	Retention time (min)	Molecular ion (m/z)	Area of peak	Stigmasterol concentration (μM)	Name of product
WT	26.78	484	2,369,453	52 μM	Stigmasta-5,22-dien-3-ol
*CYP710C1/NEO*	26.77	484	1,842,471	41 μM	Stigmasta-5,22-dien-3-ol

We also assessed if reduced expression of *CYP710C1* compromised the cellular growth of single knockouts. Growth kinetics was studied. Heterozygotes parasites exhibited delayed growth as compared to wild-type parasites (Fig 7H). Our data suggest that the *LdCYP710C1* gene has a significant role in the growth and infectivity of amastigotes.

### Heterozygous *CYP710C1 L* .*donovani* mutants have altered sterols composition

The sterol compositions of wild-type and heterozygous *CYP710C1* mutant (*CYP710C1/NEO) L*. *donovani* promastigotes strains are shown in Table 6 and Table 7. Typically, the significant sterols found in promastigotes of *L*. *donovani* were cholesterol (40%) and ergosterol (48%). In contrast, in *CYP710C1* heterozygous (*CYP710C1/NEO)* promastigotes, the significant sterols were a mixture of cholesterol (~23%) and ergosterol (~52%). No stigmasterol was observed in these mutant promastigotes. The sterol composition of axenic amastigotes cells derived from the wild-type and *CYP710C1/NEO L*. *donovani* promastigotes was also examined. The ergosterol contents in axenic amastigotes were low (~12%) when compared to wild-type promastigotes. The axenic amastigotes of wild-type cells contain ~ 42% cholesterol, ~12.5% ergosterol, ~10% stigmast-5-en-3-ol and ~13% stigmast-5, 22-diene-3-ol of total sterols (Table 7). On the contrary, the axenic amastiogotes of *CYP710C1/NEO* cell lines contained only ~1.7% of stigmasterol. This data indicated that heterozygous mutant of *CYP710C1* had altered sterol levels in *L*. *donovani*.

### *CYP710C1* overexpression results in increased resistance to AmB

**Fig 8:**
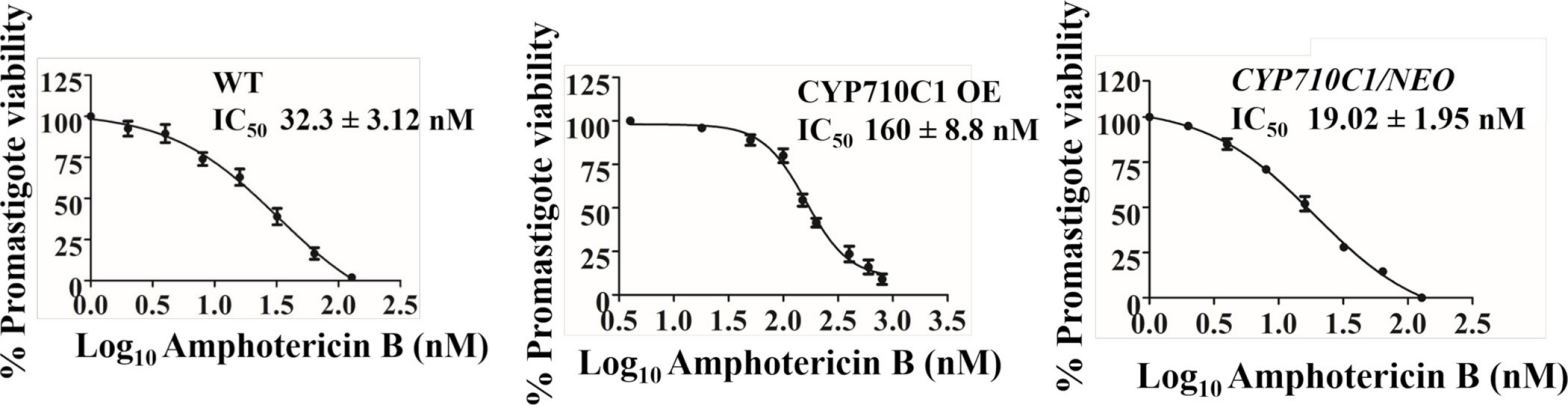
Effect of AmB on WT, CYP710C1 OE, and *CYP710C1/NEO* parasites. *In vitro* drug sensitivity was measured by incubating promastigotes at a cell density of 2x 10^5^ cells/ml in M199 medium supplemented with 10% FBS with a range of different concentrations of the AmB in 96 well plates. After 72 h, the viability of the cells was determined by the 50% inhibitory concentration (IC_50_) with the MTT assay. This value corresponds to the concentration of AmB which results in 50% inhibition of cell proliferation compared with untreated cells. CYP710C1 OE showed resistance to strains to anti-leishmanial drug AmB. CYP710C1 heterozygous *L*. *donovani* promastigotes display increased sensitivity to AmB. IC_50_ are given as mean **±** S.D of at least two independent determinations with triplicates in each.

As sterol composition is a significant determinant for AmB action, we finally asked whether overexpression of *CYP710C1* alters the resistance of the parasite to this drug. MTT assays showed that CYP710C1 OE was ~5-fold more resistant to AmB as compared to wild-type (Fig 8). However, there was no significant change in the sensitivity profile of CYP710C1 OE promastigotes to potassium antimonyl tartrate trihydrate (PAT) and miltefosine, suggesting that the increase stigmasterol composition is responsible for resistance to AmB (Table 9). We also checked the susceptibility of *CYP710C1* heterozygous *L*. *donovani* promastigotes to AmB. The heterozygous mutant strain displayed increased sensitivity to AmB. Heterozygous promastigotes (*CYP710C1/NEO*) and WT promastigotes showed that *CYP710C1/NEO* was ~1.6 -fold more sensitive to AmB as compared to that of WT (Fig 8).

**Table 9:** Drug sensitivity of wild, CYP710C1 OE and *CYP710C1/NEO* promastigotes. Values were determined in promastigotes of wild-type, CYP710C1 OE and *CYP710C1/NEO* of *L*. *donovani* after 72 h of drug addition. IC_50_ are given as mean **±** S.D of at least two independent determinations with triplicates in each. *nd- not determined.

Drug	Mean IC_50_ ± SD [μM]		
	WT	CYP710C1 OE	*CYP710C1/NEO*
Amphotericin B	0.032 ± 0.003	0.16 ± 0.008	0.019 ± 0.001
Pentamidine	0.36 ± 0.015	0.5 ± 0.025	nd
Miltefosine	10.76 ± 1.98	10.30 ± 1.5	nd
Sb^III^ (PAT)	38.95 ± 3.5	36.75 ± 3.1	nd

## Discussion

Leishmaniasis is a significant public health problem in several parts of the tropical and subtropical world. The disease treatment entirely relies on a limited repertoire of anti-leishmanial chemotherapy. However, the associated toxicity and the emergence of drug resistance have necessitated the identification of new drugs and drug targets [1].

In recent years, AmB has gained popularity as the first line treatment for leishmaniasis, particularly in its liposomal formulation. AmBisome has several advantages over non-liposomal formulation such as less toxicity, a lower dose (single dose) and no prolonged hospitalization [33].

We report here the presence of a *CYP710C1* P450 family gene that encodes a plant-like sterol C-22 desaturase, leading to stigmasterol biosynthesis in *Leishmania*. Cytochrome P450 (CYP) is a superfamily of heme-containing monooxygenases that is involved in the metabolism of endogenous or xenobiotic compounds. We reported earlier that different classes of CYPs (CYP51E1, CYP710C1, CYP5123A1 and CYP5122A1) exist in various strains/ species of *Leishmania* and *Trypanosomes* [17].

Multiple sequence alignment of CYP710C1 protein of *L*. *donovani* with homologous sequences from other strains revealed that it shares 72% with *T*. *cruzi* (CYP710C1), 40% with *Selaginella moellendorffii* (CYP710A21v1) and 38–39% identity with *Arabidopsis thaliana* (CYP710A1-4). The motifs unique to CYP719C10 are highly conserved amongst the various organisms. Despite the high degree of conservation, we were able to identify few discernible differences between CYP710C of various species; however, further studies would reveal the significance of these differences.

CYP710C1 is an NADPH-dependent C-22 desaturase that catalyses the conversion of β-sitosterol into stigmasterol. The gene present in *L*. *donovani* encodes a functional protein capable of catalyzing this reaction. Further, the expression of CYP710C1 is higher in amastigotes as compared to promastigotes, suggesting that stigmasterol may confer some benefit to the parasite when it is resident in the host macrophage.

Here we have shown the sterol composition of WT, CYP710C1 OE and *CYP710C1/NEO* promastigotes and axenic amastigotes have dramatic differences among sterol levels (Table 6 & Table 7). Overexpression of *CYP710C1* results in higher level of stigmasterol in promastigotes as compared to their respective wild-type. The percentage of stigmasterol in overexpressing promastigote was about ten times higher than the wild-type promastigotes. The promastigotes of wild-type cells revealed ~40% cholesterol, ~48% ergosterol, and ~2% stigmast-5-en-3-ol of total sterols. The CYP710C1 OE cell lines contributed 33% cholesterol, ~42% ergosterol, ~21% stigmasterol and ~17% β-sitosterol-3 acetate. In the case of *CYP710C1* heterozygous (*CYP710C1/NEO)* promastigotes, the significant sterols were a mixture of cholesterol (~23%) and ergosterol (~52%). No stigmasterol was observed in these mutant promastigotes. Furthermore, ergosterol contents in axenic amastigotes were low (~12%) when compared to wild-type promastigotes. The axenic amastigotes of wild-type cells contain ~ 42% cholesterol, ~12.5% ergosterol, ~10% stigmast-5-en-3-ol and ~13% stigmast-5, 22-diene-3-ol of total sterols (Table 7). On the other hand, the axenic amastiogotes of *CYP710C1/NEO* cell lines contained only ~1.7% of stigmasterol.

Sterols are considered as membrane reinforcers as they help to sustain the domain structure of cell membranes. Sterols are critical for the formation of liquid-ordered (lo) membrane states (lipid “rafts”) that are supposed to play an essential role in fundamental biological processes such as signal transduction, cellular sorting, cytoskeleton reorganization and asymmetric growth [34]. They have been proposed as critical molecules to maintain membranes in a state of fluidity adequate for function. A higher level of stigmasterol within plant plasma membrane has been correlated with altered fluidity and permeability characteristics [35, 36]. The sterol composition is different in CYP710C1 OE as compared to WT, possibly resulting in altered membrane integrity and membrane dynamics in CYP710C1 OE. There are reports showing parasites with altered sterols composition respond differently to anti-leishmanial drugs [15, 37].

Amphotericin B is known to mediate changes in the membrane permeability thereby inducing a leakage of important cellular constituents and ultimately lysis and death of the cell. They promote such an effect because of the presence of sterols in the cell membrane. The polyene antibiotics and sterols are reported to form molecular complexes to create channels or solid patches that disrupt the membrane [38]. The actual mechanism of action of AmB may be more complex and multifaceted.

The basis of the antifungal and antileishmanial selective toxicity of AmB is proposed to be due to the prevalence of ergosterol on the surface membranes of these organisms and a significant interaction between AmB and ergosterols. AmB binding to ergosterol leads to disruption of the osmotic integrity of the membrane in target cells and is a potent agent used for the treatment of leishmaniasis [21]. Some standard features have been detected in AmB resistant *Leishmania* such as changes in sterol metabolism, where ergosterol, the primary sterol of wild-type *Leishmania* cell membranes is reduced or lost and replaced by different cholestane-type sterols [33]. However, in mammalian cells the principal sterol is cholesterol.

Interestingly, overexpression of *CYP710C1* led to ~5-fold increase in resistance to AmB in contrast to *CYP710C1/NEO* which showed ~1.6 -fold more sensitivity to AmB as compared to the wild-type (WT), suggesting that this protein is possibly involved in modulation of target of AmB in *Leishmania*, and therefore, is possibly essential for the parasite to withstand the drug. The precise mechanism by which stigmasterol regulates membrane properties in *Leishmania* is not yet understood. Further studies are required to check the exact role of stigmasterol in AmB resistance. The presence of stigmasterol, ergosterol and their corresponding isoforms in *L*. *donovani* indicates that *Leishmania* has acquired both the fungus and plant pathways for sterol biosynthesis. The present study will offer the ability to monitor the emergence and spread of resistance to AmB drug in the field.

## Supporting information

S1 Table CT values for *CYP710C1* and *JW* gene in promastigotes and amastigotes. (DOCX)

## References

[1] Freitas-Junior LH, Chatelain E, Kim HA, Siqueira-Neto JL (2012) Visceral leishmaniasis treatment: What do we have, what do we need and how to deliver it? Int J Parasitol Drugs Drug Resist 2: 11–191 10.1016/j.ijpddr.2012.01.003 24533267 PMC3862432

[2] Garcia-Hernandez R, Manzano JI, Castanys S, Gamarro F (2012) Leishmania donovani develops resistance to drug combinations. PLoS Negl Trop Dis 6: e1974 10.1371/journal.pntd.0001974 23285310 PMC3527373

[3] Kelly DE, Krasevec N, Mullins J, Nelson DR (2009) The CYPome (Cytochrome P450 complement) of Aspergillus nidulans. Fungal Genet Biol 46 Suppl 1: S53–61. 18824241 10.1016/j.fgb.2008.08.010

[4] Morikawa T, Mizutani M, Aoki N, Watanabe B, Saga H, et al (2006) Cytochrome P450 CYP710A encodes the sterol C-22 desaturase in Arabidopsis and tomato. Plant Cell 18: 1008–1022 10.1105/tpc.105.036012 16531502 PMC1425849

[5] Moktali V, Park J, Fedorova-Abrams ND, Park B, Choi J, et al (2012) Systematic and searchable classification of cytochrome P450 proteins encoded by fungal and oomycete genomes. BMC Genomics 13: 525 10.1186/1471-2164-13-525 23033934 PMC3505482

[6] Berriman M, Ghedin E, Hertz-Fowler C, Blandin G, Renauld H, et al (2005) The genome of the African trypanosome Trypanosoma brucei. Science 309: 416–422. 10.1126/science.1112642 16020726

[7] Nelson D R (2009) The cytochrome p450 homepage *Hum Genomics* 4 59–65 10.1186/1479-7364-4-1-59 19951895 PMC3500189

[8] El-Sayed NM, Myler PJ, Bartholomeu DC, Nilsson D, Aggarwal G, et al (2005) The genome sequence of Trypanosoma cruzi, etiologic agent of Chagas disease. Science 309: 409–415. 10.1126/science.1112631 16020725

[9] El-Sayed NM, Myler PJ, Blandin G, Berriman M, Crabtree J, et al (2005) Comparative genomic of trypanosomatid parasitic protozoa. Science 309: 404–409. 10.1126/science.1112181 16020724

[10] Ivens AC, Peacock CS, Worthey EA, Murphy L, Aggarwal G, et al (2005) The genome of the kinetoplastid parasite, Leishmania major. Science 309: 436–442. 10.1126/science.1112680 16020728 PMC1470643

[11] McCall LI, El Aroussi A, Choi JY, Vieira DF, De Muylder G, et al (2015) Targeting Ergosterol biosynthesis in Leishmania donovani: essentiality of sterol 14 alpha-demethylase. PLoS Negl Trop Dis 9: e0003588 10.1371/journal.pntd.0003588 25768284 PMC4359151

[12] Galea AM, Brown AJ (2009) Special relationship between sterols and oxygen: were sterols an adaptation to aerobic life? Free Radic Biol Med 47: 880–889 10.1016/j.freeradbiomed.2009.06.027 19559787

[13] Goad LJ, Holz GG Jr., Beach DH (1984) Sterols of Leishmania species. Implications for biosynthesis. Mol Biochem Parasitol 10: 161–170. 6700638 10.1016/0166-6851(84)90004-5

[14] Schaller H (2003) The role of sterols in plant growth and development. Prog Lipid Res 42: 163–175. 12689617 10.1016/s0163-7827(02)00047-4

[15] Benveniste P (2004) Biosynthesis and accumulation of sterols. Annu Rev Plant Biol 55: 429–457. 10.1146/annurev.arplant.55.031903.141616 15377227

[16] Vranova E, Coman D, Gruissem W (2013) Network analysis of the MVA and MEP pathways for isoprenoid synthesis. Annu Rev Plant Biol 64: 665–70. 10.1146/annurev-arplant-050312-120116 23451776

[17] R Bansal HS, VS Gowri, SS Sen, I Ghosh, R Madhubala (2017) The Cytochrome P450 Complement (CYPome) of Leishmania leads to the discovery of a plant like Cytochrome P450 subfamily CYP710C1 gene. Proceedings of the indian national science academy Vol 83 No 3 .

[18] Desmond E, Gribaldo S (2009) Phylogenomics of sterol synthesis: insights into the origin, evolution, and diversity of a key eukaryotic feature. Genome Biol Evol 1: 364–381. 10.1093/gbe/evp036 20333205 PMC2817430

[19] Bach TJ RM (2012) Isoprenoid Synthesis in Plants and Microorganisms: New Concepts and Experimental Approaches.

[20] Kelly SL, Kelly DE (2013) Microbial cytochromes P450: biodiversity and biotechnology. Where do cytochromes P450 come from, what do they do and what can they do for us? Philos Trans R Soc Lond B Biol Sci 368: 20120476 10.1098/rstb.2012.0476 23297358 PMC3538425

[21] Palacios DS, Dailey I., Siebert D.M., Wilcock B.C., and Burke M.D. ((2011).) Synthesis-enabled functional group deletions reveal key underpinnings of amphotericin B ion channel and antifungal activities. Proc. Natl. Acad. Sci. U. S. A. 108, 6733–6738. 10.1073/pnas.1015023108 21368185 PMC3084054

[22] Gray KC, Palacios DS, Dailey I, Endo MM, Uno BE, et al (2012) Amphotericin primarily kills yeast by simply binding ergosterol. Proc Natl Acad Sci U S A 109: 2234–2239. 10.1073/pnas.1117280109 22308411 PMC3289339

[23] Chattopadhyay A, Jafurulla M (2011) A novel mechanism for an old drug: amphotericin B in the treatment of visceral leishmaniasis. Biochem Biophys Res Commun 416: 7–12. 10.1016/j.bbrc.2011.11.023 22100811

[24] Geraghty P, Kavanagh K (2003) Disruption of mitochondrial function in Candida albicans leads to reduced cellular ergosterol levels and elevated growth in the presence of amphotericin B. Arch Microbiol 179: 295–30. 10.1007/s00203-003-0530-y 12640519

[25] Trajkovic V, Markovic M, Samardzic T, Miljkovic DJ, Popadic D, et al (2001) Amphotericin B potentiates the activation of inducible nitric oxide synthase and causes nitric oxide-dependent mitochondrial dysfunction in cytokine-treated rodent astrocytes. Glia 35: 180–188. 11494409 10.1002/glia.1083

[26] Debrabant A, Joshi MB, Pimenta PF, Dwyer DM (2004) Generation of Leishmania donovani axenic amastigotes: their growth and biological characteristics. Int J Parasitol 34: 205–217. 10.1016/j.ijpara.2003.10.011 15037106

[27] Heidi J.,Halbwirth H., and Oliver S. (2018) Recombinant production of eukaryotic cytochrome P450s in microbial cell factories. Bioscience Reports. 10.1042/BSR20171290. PMC5835717 29436484

[28] Chou Chin-Cheng & Liu Yu-Pin (2004) Determination of fecal sterols in the sediments of different wastewater outputs by GC-MS, International Journal of Environmental Analytical Chemistry, 84:5, 379–388, 10.1080/03067310410001680019

[29] Mosmann T (1983) Rapid colorimetric assay for cellular growth and survival: application to proliferation and cytotoxicity assays. Journal of Immunological Methods 65: 55–63. 6606682 10.1016/0022-1759(83)90303-4

[30] Kapler GM, Coburn CM, Beverley SM (1990) Stable transfection of the human parasite Leishmania major delineates a 30-kilobase region sufficient for extrachromosomal replication and expression. Mol Cell Biol 10: 1084–1094. 2304458 10.1128/mcb.10.3.1084-1094.1990 PMC360971

[31] Sambrook J, Fritsch EF, Maniatis T (1989) Molecular cloning: a laboratory manual: Cold spring harbor laboratory press.

[32] Rakotomanga M, Blanc S, Gaudin K, Chaminade P, Loiseau PM (2007) Miltefosine affects lipid metabolism in Leishmania donovani promastigotes. Antimicrob Agents Chemother 51: 1425–1430. 10.1128/AAC.01123-06 17242145 PMC1855451

[33] Mwenechanya R, Kovarova J, Dickens NJ, Mudaliar M, Herzyk P, et al (2017) Sterol 14alpha demethylase mutation leads to amphotericin B resistance in Leishmania mexicana. PLoS Negl Trop Dis 11: e0005649 10.1371/journal.pntd.0005649 28622334 PMC5498063

[34] Simons K, Ehehalt R. Cholesterol, lipid rafts, and disease. J Clin Invest. 2002;110:597–60 10.1172/JCI16390 12208858 PMC151114

[35] Arnqvist L., Persson M., Jonsson L., Dutta P. C., and Sitbon F. (2008) Overexpression of CYP710A1 and CYP710A4 in transgenic Arabidopsis plants increases the level of stigmasterol at the expense of sitosterol. *Planta* 227, 309–317 10.1007/s00425-007-0618-8 17909855

[36] Wang K., Senthil-Kumar M., Ryu C. M., Kang L., and Mysore K. S. (2012) Phytosterols play a key role in plant innate immunity against bacterial pathogens by regulating nutrient efflux into the apoplast. *Plant Physiol* 158, 1789–1802 10.1104/pp.111.189217 22298683 PMC3320186

[37] Mathur R, Das RP, Ranjan A, Shaha C (2015) Elevated ergosterol protects Leishmania parasites against antimony-generated stress. FASEB J 29: 4201–4213. 10.1096/fj.15-272757 26116701

[38] Purkait B, Kumar A, Nandi N, Sardar AH, Das S, Kumar S, et al Mechanism of amphotericin B resistance in clinical isolates of *Leishmania donovani*. Antimicrob Agents Chemother. 2012; 56(2):1031± 41. 10.1128/AAC.00030-11 22123699 PMC3264217

