# Supplementary material for: A Plant like Cytochrome P450 Subfamily CYP710C1 Gene in Leishmania donovani Encodes Sterol C-22 Desaturase and its Over-expression Leads to Resistance to Amphotericin B
Source: PLoS Negl Trop Dis. 2019 Apr 3;13(4):e0007260. doi: 10.1371/journal.pntd.0007260 (PMC6464246; doi:10.1371/journal.pntd.0007260)
Supplement: S1 Table — (DOCX) [file pntd.0007260.s001.docx]

**S1 Table.** **C_T_ values for CYP710C1 and JW gene in promastigotes and amastigotes.**

| **Gene** | **C_T_ value** | |
| --- | --- | --- |
|  | **Promastigotes** | **Amastigotes** |
| **CYP710C1 gene** | **24.696 ± 0.50** | **29.853 ± 0.11** |
| **kDNA minicircle gene ( JW )** | **26 ± 0.12** | **32.403 ± 0.53** |
